# Supplementary material for: End of treatment and 12-month post-treatment outcomes in patients treated with all-oral regimens for rifampicin-resistant tuberculosis in Ukraine: a prospective cohort study
Source: PLOS Glob Public Health. 2025 May 23;5(5):e0003983. doi: 10.1371/journal.pgph.0003983 (PMC12101767; doi:10.1371/journal.pgph.0003983)
Supplement: S1 Table — (DOCX) [file pgph.0003983.s001.docx]

**Table S1. Type of drugs with permanent change (interruption >30 days) among RR-TB patients in Zhytomyr Oblast, Ukraine, April 2019 – March 2022**

|  | **Total (n=95)** |
| --- | --- |
| **TB drug** | **n (%)** |
| Levofloxacin | 58 (61) |
| Linezolid | 14 (15) |
| Cycloserine | 9 (10) |
| Delamanid | 5 (5) |
| Bedaquiline | 4 (4) |
| Terizidone | 3 (3) |
| Clofazimine | 2 (2) |
