# Supplementary material for: End of treatment and 12-month post-treatment outcomes in patients treated with all-oral regimens for rifampicin-resistant tuberculosis in Ukraine: a prospective cohort study
Source: PLOS Glob Public Health. 2025 May 23;5(5):e0003983. doi: 10.1371/journal.pgph.0003983 (PMC12101767; doi:10.1371/journal.pgph.0003983)
Supplement: S2 Table — (DOCX) [file pgph.0003983.s002.docx]

**Table S2. Reason of permanent drug changes (interruption >30 days) among RR-TB patients in Zhytomyr Oblast, Ukraine, April 2019 – March 2022**

|  | **Total (n=95)** |
| --- | --- |
| **Reason of permanent drug changes** | **n (%)** |
| Resistance to drug | 52 (55) |
| Adverse event | 13 (14) |
| Planned change | 9 (10) |
| Reintroduction/replacement of stopped drug | 4 (4) |
| Drug supply or drug administration issues | 4 (4) |
| Other reason | 5 (5) |
| Unknown | 8 (8) |
