# Supplementary material for: End of treatment and 12-month post-treatment outcomes in patients treated with all-oral regimens for rifampicin-resistant tuberculosis in Ukraine: a prospective cohort study
Source: PLOS Glob Public Health. 2025 May 23;5(5):e0003983. doi: 10.1371/journal.pgph.0003983 (PMC12101767; doi:10.1371/journal.pgph.0003983)
Supplement: S3 Table — (DOCX) [file pgph.0003983.s003.docx]

**Table S3. Outcome of patients with at least one permanent change on their regimen (interruption >30 days) among RR-TB patients in Zhytomyr Oblast, Ukraine, April 2019 – March 2022**

|  | **Total (n=79)** |
| --- | --- |
| **Outcome** | **n (%)** |
| Cured | 59 (75) |
| Completed | 8 (10) |
| Died | 7 (9) |
| Failed | 2 (2) |
| LTFU | 3 4) |
