# Supplementary material for: End of treatment and 12-month post-treatment outcomes in patients treated with all-oral regimens for rifampicin-resistant tuberculosis in Ukraine: a prospective cohort study
Source: PLOS Glob Public Health. 2025 May 23;5(5):e0003983. doi: 10.1371/journal.pgph.0003983 (PMC12101767; doi:10.1371/journal.pgph.0003983)
Supplement: Attachment — Submitted filename: Response to Reviewers.docx [file pgph.0003983.s007.docx]

**Reviewer #1:**

Overall, this is a well conducted observational study assessing person level outcomes for the treatment of MDR/RR-TB using oral, short treatment regimens. Given the difficult context in which the study was conducted, these data are important to demonstrate how such treatment can be provided and good outcomes achieved. The study is therefore relevant to a wide reader audience.
We thank the reviewer for their kind and supporting comment.

*Major comment:*
The manuscript would be greatly improved by following STROBE guidance for presentation of observational studies; see <https://www.equator-network.org/reporting-guidelines/strobe/> This would include clearer presentation of the baseline characteristics of the study cohort and a flow diagram describing eligibility, inclusion and exclusion for the study.
We have indeed used the STROBE guidance and added the checklist. We uploaded the filled checklist accompanying our revision submission. We did not provide a flow diagram since this was a single arm study where consecutive patients who met the reported eligibility criteria were recruited for a very specific period due to logistics/supply limitations regarding the drugs included in the regimen.

Other comments:
• Lines 108-109: there is mention of treatment education, but a much more detailed description is given later – perhaps to exclude here?

The phrase is now deleted.

• Lines 110-112: the section describing the provision of ambulatory care as different from national policy could be clarified.

We explained that while the national policy required hospitalization for the entire duration of the treatment, our approach was to discharge patients once they achieved culture conversion to negative.

• Line 147: how was adherence assessed?

Adherence throughout the treatment was assessed by a multidisciplinary team as described in the paragraph. This was discussed between the patient and the assigned counsellor and to

specific persons we offered the option of video-observed treatment (VOT). We did not use laboratory-based methods.

• Define acronyms at first use, eg. PWID

We have now defined missing acronyms

• Box 1: How was the community engaged to provide support to patients and families?

Throughout the intervention we engaged with community NGOs while also conducting health promotion activities.

• Under results, there appear to be 2 sections titled “end of treatment outcomes”.

We think it might be an error while transforming to PDF? In our word manuscript we have one end of treatment outcomes section (the other on is on 12 month outcomes)

• Please revise language to refer to ‘treatment failure” rather than “failed”, which implies that the individuals themselves have failed in some way.

WE ABSOLUTELY AGREE and thank the reviewer for highlighting this lapsus. We have corrected this.

• Were any post-treatment outcomes available for people who had treatment outcomes of “treatment failure” or LTFU? I see this is described in the discussion, but could be presented in the results.

We only followed up patient who were cured or completed their treatment. In Discussion we only discuss about those patients.

• For table 5, please add a description of each scenario to the table legend.

This is now added.

• For the time to culture conversion analysis, was it not possible to censor for death and LTFU? Rather than exclude these patients completely? Additionally, you report time to culture conversion by alcohol use, but no other factors; were any other relevant factors significant? Eg. extent of drug resistance or previous TB treatment?

We perfectly understand the suggestion for censoring instead of excluding. However, neither censoring not excluding account for the fact that death and loss to follow up are “competing risks” and therefore the assumptions for typical survival analysis would not be met. An alternative analytical approach would have been to estimate “cumulative incidence” however after seeking statistical advice and looking at the existing literature where cumulative incidence is rarely, if ever, reported in such studies we decided to drop the survival analysis which in any case did not offer substantial information. We kept, however, the reporting of the median time to culture conversion among all people who had a baseline positive culture. We have similarly removed the specific analysis by AUD.

The following article clearly describes the lack of consensus regarding such analyses and the lack of clarity and transparency in reporting.

**Sputum culture conversion definitions and analytic practices for multidrug-resistant TB**

<https://pmc.ncbi.nlm.nih.gov/articles/PMC8259120/#:~:text=Conversion%20of%20sputum%20culture%20from,RR%2FMDR%2DTB).&text=Sputum%20culture%20conversion%20is%20also,outcome%20in%20observational%20cohort%20studies>.

• Perhaps to clarify why pregnancy during treatment might be considered an adverse event?

We agree that this part was confusing. There was no unwanted pregnancy recorded in any case. We have now deleted this phrase from Table 7.

• The methods mention deep sequencing, but no data pertaining to this is presented. What was the purpose of deep sequencing of TB isolates?

Thank you for pointing this out. Our initial plan was to use sequencing to differentiate between re-infection and recurrence. We also thought of contributing data to the national and global epidemiological studies. However due to resource constrains we omitted this part. WE have deleted the phrase from the methods section.

• To clarify that the majority of patients would have completed treatment by the time of the Russian invasion of Ukraine in Feb 2022 – does this mean that the modified treatment provisions were perhaps only relevant for the post-treatment period for most patients?

This is correct. Most of the patients had their treated completed before the invasion, however the post-treatment follow up which was a main aim of our study (sustained treatment response) was done almost entirely during the invasion and despite patients and our MSF & MOH teams on the ground being directly and indirectly affected by the conflict. Zhytomyr was affected particularly at the early stages of the conflict. We were deeply moved by the dedication of our colleagues who remained in the area to continue with the care of the patients and the collection of data for the study completion, during such hard and dangerous times.

**Reviewer #2:**

*General Comments:*
The treatment of rifampin-resistant TB (RR-TB) has changed dramatically over the past 10 years—most notably with the change from 18-24 month injectable-based regimens to 6-9 month all-oral regimens incorporating new and repurposed medications. Many prior cohort studies have reported RR-TB treatment outcomes from a variety of programmatic settings around the world. Context is often key, however, when interpreting and comparing results from different countries as some settings lack access to newer diagnostics or medications, while others may partner with NGOs to provide additional patient-centered support.

In this manuscript, authors from Medecins Sans Frontieres report their experience supporting an RR-TB program in Ukraine over a time period spanning both the emergence of the COVID pandemic and the start of the war with Russia.

Clearly, the real-life challenges to providing (and completing) RR-TB care in this setting are vast, yet the authors demonstrate that they were able to achieve startlingly favorable end-of-treatment outcomes. The paper is very well-written and clear (though there are a number of typographical and formatting errors). Importantly, the authors emphasize not only the mechanics of providing treatment, but also of supporting patients socially to ensure treatment completion. Participants were administered quality-of-life questionnaires. Of particular value is that the authors also captured post-treatment outcomes 12-months after treatment completion. They note high rates of LTFU in this post-treatment period and raise the possibility that relapse/death could be much higher in the long-term, even if end-of-treatment outcomes were favorable. The data are a valuable contribution to the literature and should serve as a model to others trying to set up or maintain RR-TB services in conflict-areas.

We thank the reviewer for their kind and supporting comment.

*Specific Comments:*

ABSTRACT
1) Line 47, Results: Suggest delete the word “and.”
Deleted

INTRODUCTION
1) Why are dates of enrollment are highlighted throughout the manuscript?

Apolofies for this. We highlighted the dates during the pre-review checks to show the consistency between different parts of the manuscript. We have now removed the highlight.

2) Line 98: “prevalence…” Suspect that the authors mean “incidence” here.

We believe that the term here is correct.

3) Line 100: Would consider using a word other than “Dispensary.” Although this is what they are called in Ukraine, the term “dispensary” in the rest of the world more often refers to a pharmacy. Could say “TB referral hospital” or “TB specialist hospital/center” instead.

We agree. Thank you for this remark. We have now changed to hospital

4) Line 100: “The dispensary also coordinated the ambulatory care…” How so? Do patients go there for outpatient care or does the dispensary arrange for patients to be seen at some decentralized clinic instead?

We have now added text to explain how the hospital coordinated the ambulatory part of care across different type of facilities and providers.

5) Line 114: “…outpatient facilities…” See comment above. How close are these facilities? Are they spread out over a wide geographic area? Close to patients’ homes?
Thank you for this comment. We have now edited this phrase to explain that the ambulatory phase was organized throughout the oblast and in close proximity to patient’s homes. We have assessed in fact all available facilities and studied their preparedness. We publish the assessment and reference the publication.

METHODS
1) No methods are provided for the logistic regression presented in the Results. How were covariates selected for the multivariable model?
Thank you for this very helpful comment; we realized that this part of the analysis was written in the Results instead of the Methods. We have now added the following paragraph in the statistical analysis part of the Methods.
*We included variables with a p-value <0.2 in the initial multivariable model, as well as factors that made sense clinically, even if their p-value was slightly above 0.2. We then refined the model using backward elimination and stepwise selection.*

RESULTS
1) Line 244: “End…” is not bolded while the rest of the heading is.

Corrected
2) Line 247: “(Table & 3)”: Typo

Corrected
3) Lines 250-1: The placement of the percentages and p-values in the sentences is confusing.

We have no changed from semi-colon to comma as more appropriate to separate percentages from p-value).
4) Lines 255-6: “Of our patients, 233 (78%) were male, had a median age of 45 years…” Grammatical error. Would rephrase.

Edited. “From 1 April 2019 to 31 May 2021, 300 patients were enrolled in the study with a median age of 45 years (IQR 38-52 years). Table 1 shows the key characteristics of these patients. Among these, 233 (78%) were male, 69 (23%) were living with HIV, 6 (2%) had Hepatitis B, 60 (20%) with history or active Hepatitis C (HCV), 13 (4%) had diabetes mellitus, 204 (69%) smoked cigarettes, 14 (5%) persons who injected drugs (PWID), 68 (24%) had hazardous drinking, and 73 (25%) had alcohol dependence. “
5) Line 258: Would avoid the non-person-centered term “smoker” (suggest “smoked cigarettes”).

Edited.
6) Line 260: “Error! (Reference source not found)”—Likely typographical error.

Corrected
7) Line 261-269: This paragraph appears to have been completely duplicated ( lines 244-252)

Corrected
8) Line numbers start again from 1 halfway through the RESULTS.

This is happening due to the page break structure to fit the tables. We tied to resolve but it might recur.
9) Second line 4: Suggest “…with a median time to death of 7.9 months…”

Edited

10) Second lines 34-6: “…45 died or were LTFU before six months and were excluded from the 6-month culture-conversion analysis.” There are many different philosophies about the best approach in the longitudinal analysis of culture conversion. Excluding those who die or who are LTFU clearly introduces a survival bias to even be eligible for the analysis. One could argue that Death prior to culture conversion is, itself, strongly associated with NOT converting. That said, not everyone who is LTFU necessary fails to convert (just as not everyone who dies does so from TB) so including them in the analysis may negatively skew the results. Regardless, however, the authors should simply provide a clear justification of their approach and explain why their approach will minimize certain biases while acknowledging that it may introduce others.

We perfectly understand and agree with these comments. An alternative approach we considered (and was suggested by another reviewer) was for censoring instead of excluding. However, neither censoring nor excluding would have appropriately account for the fact that death and loss to follow up are competing risks and therefore the assumptions for typical survival analysis would not have been met. An alternative analytical approach would have been to estimate “cumulative incidence” however after seeking statistical advice and looking at the existing literature where cumulative incidence is rarely, if ever, reported in such studies we decided to drop the survival analysis which in any case did not offer substantial information. We kept, however, the reporting of the median time to culture conversion among all people who had a baseline positive culture. We have similarly removed the specific analysis by AUD.

The following article clearly describes the lack of consensus regarding such analyses and the lack of clarity and transparency in reporting.

**Sputum culture conversion definitions and analytic practices for multidrug-resistant TB**

<https://pmc.ncbi.nlm.nih.gov/articles/PMC8259120/#:~:text=Conversion%20of%20sputum%20culture%20from,RR%2FMDR%2DTB).&text=Sputum%20culture%20conversion%20is%20also,outcome%20in%20observational%20cohort%20studies>.

11) Second line 49: Would suggest providing the number of participants as well as the total number of AEs.

Corrected
12) Second Line 52: “experience” should be “experienced.”

Corrected
13) Second Line 53: “The full list of…” Sentence appears to have been truncated erroneously.

We cannot see this. It might be an error.
14) Second Line 54: “Table.” (Is there a table missing? Not numbered. Typo?)
We cannot see this. It might be an error.

DISCUSSION
1) Second line 117: Suspect that the authors mean “serious” rather than “severe.”
Edited

TABLES/FIGURES/BOXES
Box 1:
1) The verb tense varies in the “expected outcomes” column (e.g., “Patients realize….” vs. “Patients learned….” vs. “Family members understand….” Would encourage consistent use of one tense (probably present or future if this an “expected” rather than observed outcome).

This has been edited
2) Define abbreviations in the Box (AUD, SUD, VOT, SAT, etc).

Abbreviations defined.
3) In the last section (“Community Support”), part of “Involving communities” is bolded in error.

Edited

Table 1
4) See earlier comment about the term “smoker.”
Edited
Table 2
5) The number 6 in the footnotes should be a superscript.
Edited
Table 6
6) How were characteristics selected for inclusion in the multivariable analysis? Three of the four included covariates had a p-value >0.1 even in bivariate analysis.

As we explained above, we have now added the following paragraph in the statistical analysis part of the Methods.
*We included variables with a p-value <0.2 in the initial multivariable model, as well as factors that made sense clinically, even if their p-value was slightly above 0.2. We then refined the model using backward elimination and stepwise selection.*

Table 7
7) Second line 60: Footnote 2: Suggest deleting the word “which.”

Edited
8) Second line 62: Suggest deleting the first clause and beginning with “Defined as the decision…”

Edited
9) In this table it says that 79 participants had a permanent change in regimen due to AEs, but in supplemental table S2 this number is only 13.

This is the correct number. The figures in S1-3 were miscalculated. We apologize for this and thank you for helping us identify this error.

Table 8
10) Footnotes are not provided for 1-5?

The footnotes were accidentally added. We have now deleted them.

Supplementary Table S1
11) Although mention is made in the Methods about “Permanent changes.,” the supplementary tables are not referenced anywhere in the main text.
12) Hard to interpret this table without knowing how many patients got each drug. It looks as though levofloxacin is very poorly tolerated. It might be more helpful if Tables S1 and S2 were combined so that one could see WHY each drug was stopped (I suspect that levofloxacin was stopped for resistance, while clofazimine was stopped for side effects).
13) What is the denominator for this table? Participants? Why only 95?

As described above, on a further scrutiny we were not able to verify that these supplementary tables were correctly collated. We thank you for helping us identifying this error. We have decided that to remove the Supplementary files.
